# Effect of Addition Amount on Rheological, Structural, and Sensory Properties of Whole-Grain Sweet Potato Noodles Using Extrusion

**DOI:** 10.3390/foods14061040

**Published:** 2025-03-19

**Authors:** Yan Zeng, Jie Wang, Mengxiao Bao, Yue Wu, Zhigang Chen

**Affiliations:** 1 Glycomics and Glycan Bioengineering Research Center, College of Food Science and Technology, Nanjing Agricultural University, Nanjing 210095, China; zengyan@stu.njau.edu.cn (Y.Z.); jiewxx@stu.njau.edu.cn (J.W.); a17860730179@163.com (M.B.); 2 College of Plant Protection, Nanjing Agricultural University, Nanjing 210095, China

**Keywords:** sweet potato dough, extruded sweet potato noodles, dough properties, noodles quality, microstructure

## Abstract

Whole grain foods have been recommended to preserve biologically active components and benefit human health. The effect of the addition amount of whole sweet potato flour (WSPF, 25%, 51%, and 75%) on the physicochemical and structural properties of extruded whole-grain noodles was evaluated. Compared with traditional wheat flour (WF), the increased content of WSPF led to an enhancement in the dough’s water retention capacity, resulting in the reduction of dough development time and stability time. The modulus of elasticity and the modulus of loss of the dough exhibited a positive correlation with the proportion of WSPF added, while the tangent value and maximum creep flexibility were negatively correlated. Confocal laser scanning microscopy (CLSM) observed that WSPF induced protein aggregation in the dough. Compared to conventional WF, the increased incorporation of WSPF resulted in improved textural characteristics of the extruded noodles. Sensory evaluation indicated that the addition of WSPF could enhance the quality of the noodles by imparting a sweet potato aroma, a distinctive color, and a satisfactory taste. These characteristics were correlated with their enhanced relative crystallinity, enthalpy, and short-range ordered structure. Additionally, 75% whole-grain sweet potato noodles exhibited the highest relative crystallinity (11.05%), enthalpy of pasting (Δ*H*, 22.6 J/g), and short-range ordered structure (0.78). SEM results indicated that the presence of holes in the cross-section of the sweet potato extruded noodles facilitated their rapid rehydration. Overall, the whole-grain sweet potato noodles have great potential in promoting the textural, sensory, and nutritional properties compared to traditional wheat noodles.

## 1. Introduction

Whole sweet potato flour (WSPF) is a powdered product derived from sweet potatoes through a series of processes, including washing, slicing, drying, and crushing. It retains most of the nutrients found in sweet potatoes and is rich in carbohydrates, dietary fiber, vitamins, minerals, and biologically active compounds [1]. These components contribute to energy provision, promote intestinal health, participate in physiological regulation, maintain normal body metabolism, and offer antioxidant and anticancer properties, thereby helping to prevent chronic diseases [2]. Whether used as a staple food or in various culinary preparations, WSPF is a nutritious and healthy ingredient choice. Noodle dishes, deeply rooted in the culinary traditions of numerous Asian regions, are highly valued for their wide array of shapes and the multitude of culinary methods employed in their preparation. However, WF, the raw material for traditional noodles, tends to lose significant nutrients, such as fiber, vitamins, and minerals, when the bran is removed during processing [3]. The incorporation of WSPF into noodle manufacturing has garnered considerable research attention.

Due to the absence of gluten proteins in WSPF, excessive use of this ingredient in traditional noodle production can lead to broken noodles and a cloudy soup [4]. Consequently, there is a need to explore innovative methods for manufacturing sweet potato noodles. The extrusion technique leverages the heat and mechanical stress within the extrusion equipment to form a viscous starch matrix [5]. The extrusion maturation process enhances the texture of the noodles, making them more compact and imparting a gluten-like, chewy quality. This improvement helps prevent the noodles from breaking or becoming mushy during cooking, effectively addressing the issue of insufficient gluten proteins in WSPF, which can lead to noodle breakage, muddy soup, and other problems [6].

Given the lack of a specific threshold for substituting WSPF, we adopt the criteria for whole grain foods, which require that such products consist of at least 50% whole grain by dry weight [7]. China’s definition of whole grain foods stipulates that the mass of whole grain ingredients must constitute at least 51% of the total mass of the food (on a dry basis) [8]. In line with these criteria and the framework suggested by Zhang et al. [9], our research set a benchmark substitution rate of 51% for WSPF in noodle formulation. We conducted a comprehensive analysis of how substituting WSPF at levels of 0%, 25%, 51%, and 75% affects the attributes of noodles produced through extrusion. The dough stage is pivotal in noodle manufacturing, with the mixing attributes having a substantial impact on noodle quality and manufacturability. The extent of starch gelatinization and maturation within the dough is essential for determining the textural profile of the final noodle product [10]. Dough with good flowability and ductility can pass through the die of the extruder more quickly and evenly during the extrusion process, thereby improving productivity [11]. There has not been any systematic study on the high substitution ratio of WSPF in dough, as well as in extruded ready-to-eat noodles.

In this paper, we primarily employ thermal fluidization technology to dry sweet potatoes with their skins intact, followed by ultra-micro-milling technology to micronize the treated sweet potatoes, resulting in WSPF. With WF serving as the baseline for comparison, our research explores how replacing WF with WSPF in increments of 25%, 51%, and 75% influences the dough’s mixing properties, rheological properties, and its microstructure. Moreover, the study evaluates the textural and sensory qualities of whole-grain sweet potato noodles using extrusion, in conjunction with the microstructure and starch structure of the noodles, to determine the optimal additive amount of WSPF. By systematically varying the substitution ratio, the role of WSPF in the dough can be thoroughly understood, encompassing microstructural changes and macroscopic quality performance. This involves assessing the interactions between starch and proteins, changes within the gluten matrix, as well as the properties of starch reformation. This type of evaluation reveals the impact of varying proportions of WSPF on the attributes of noodles made by extrusion processes, thereby offering a precise formulation foundation for producing extruded noodles that maintain stable quality and meet consumer demands.

## 2. Materials and Methods

### 2.1. Materials and Chemicals

WF (wheat flour for Yuanbao brand dumplings, which contains 11.0% protein, 1.6% fat, 73.5% carbohydrates, and 13.5% moisture, was purchased from IHG Food Industry Co., Ltd., Zhengzhou, China).

WSPF was prepared by cutting sweet potatoes into strips and placing them in an industrial thermal fluidization device (GW-100, Counting Time Technology Co., Ltd., Huai’an, China). The apparent gas velocity was set at 3.0 m/s, and the sweet potatoes were heated under five different temperature conditions: 110 °C, 160 °C, 150 °C, 140 °C, and 125 °C for 4 min at each stage. After drying, the sweet potato flour was processed using ultra-micronization equipment (CW150, Digital Time Technology Co., Ltd., Huai’an, China) to microfine the sweet potato chips. The flour was then passed through a 100-mesh sieve to obtain WSPF, which contained 6.7% protein, 0.46% fat, 6.0% moisture, and 14.2% dietary fiber.

The chemicals glutaraldehyde and ethanol were procured from Aladdin Biochemical Technology Co., Ltd., located in Shanghai, China. The fluorescent dyes, fluorescein isothiocyanate (FITC) and rhodamine B, were purchased from Sigma Aldrich Trading Co., Ltd., also based in Shanghai, China. The optimal cutting temperature (OCT) compound was sourced from Solarbio Technology Co., Ltd., which is headquartered in Beijing, China. It is noteworthy that all the chemical reagents used throughout this research were of the highest analytical grade.

In line with findings from prior research [9], a mixture of WSPF and WF was prepared at substitution rates of 25%, 51%, and 75%. For comparative purposes, a batch using solely WF served as the control group in the fabrication of sweet potato doughs (SPDs) and the subsequent extrusion of sweet potato noodles (ESPNs).

### 2.2. Determination of Amylose Content in WSPF and WF

Determination of total amylose contents of WSPF and WF using Solebo kits (BC0700, Beijing Solarbio Science and Technology Co., Ltd., Beijing, China).

Determination of linear amylose contents of WSPF and WF using Solebo kits (BC4260, Beijing Solarbio Science and Technology Co., Ltd., Beijing, China).

The free amylose content of WSPF was evaluated following the guidelines in Appendix A of the NY/T3611-2020 standard for “Whole Sweet Potato Flour” [12].

### 2.3. Mixolab Measurement of SPDs

The standard Chopin+ program of the Mixolab2 mixing machine (Chopin Technologies, Paris, France) was utilized to analyze the characteristics of the dough produced from the blended flour. The mixing speed during the analysis was set at 80 rpm, with initial mixing and tank temperatures of 30 °C. The target torque value for C1 was 1.1 ± 0.5 Nm. Initially, the dough underwent cooking at a temperature of 30 °C for 8 min. Subsequently, the temperature was increased to 90 °C, with a gradient of 4 °C per minute, and maintained at this level for 7 min. Afterward, the dough was cooled down to 50 °C, also at a rate of 4 °C per minute, and maintained at this temperature for an additional 5 min. This sequence of thermal treatments resulted in an overall experimental time of 45 min, as detailed by Ma et al. [13].

### 2.4. Rheological Measurements of SPDs

Based on Sun et al.’s [14] experimental protocol, performing dynamic frequency scanning and transient creep recovery scanning measurements on the dough samples utilizing an AMCR 302 rheological testing device from Anton Paar GmbH, Graz, Austria. An appropriate amount of dough was placed in the Mixolab mixing tester to achieve the target C1 torque. The dough sample was placed onto a 40 mm diameter test platform of the rotational rheometer, maintaining a 2 mm plate separation. The excess dough was trimmed after plate compression, followed by the application of silicone oil along the periphery as a sealant.

#### 2.4.1. Dynamic Frequency Sweep Test

The linear viscoelastic behavior of each dough specimen was characterized through oscillatory frequency measurements at 25 °C, with the strain amplitude fixed at 0.1%. The angular frequency (*ω*) ranged from 0.1 to 100 rad/s. These measurements enabled us to evaluate the frequency dependence of storage modulus (G′), loss modulus (G″), and damping factor (tan *δ*, calculated as G″/G′). The relationship between G′ and *ω* is modeled using a power law, as described in Equation (1)

(1) $$G^{\prime }=K{\left(\omega \right)}^{z}$$

#### 2.4.2. Creep Recovery Scanning Test

Sample viscoelastic response was evaluated at 25 °C through a two-phase analysis: an initial creep phase with 65 Pa constant stress maintained for 150 s, followed by a 300 s stress-free recovery phase.

### 2.5. Confocal Laser Scanning Microscopy (CLSM) of SPDs

The internal structure of the dough was analyzed employing a confocal laser scanning microscope (CLSM) model ZEISS LSM 980 equipped with Airyscan 2 technology, provided by Carl Zeiss, Germany. This analysis was conducted with a slight adaptation of the protocol established by Shi et al. [15]. The dough was cut into squares measuring 10 mm on each side and fixed in glutaraldehyde for 8 h. The glutaraldehyde was subsequently rinsed off with 20 mL of alcohol, followed by rinsing with 20 mL of deionized water. The samples were then embedded in OCT and sectioned to a thickness of 20 μm using a frozen slicer (CM1950, Leica Instruments, Oberkochen, Germany). The tissue samples were affixed to glass slides for microscopic examination and treated with a staining protocol involving 50 µL of a mixed solution (1:1 ratio) that included 0.25% (*w*/*w*) fluorescein isothiocyanate (FITC) and 0.025% (*w*/*w*) rhodamine B, applied for 2 min. Post-staining, the slides were washed with 100 µL of purified water to remove excess stain. The CLSM was utilized to obtain images at a pixel dimension of 1024 × 1024, utilizing excitation wavelengths of 488 nm for FITC and 561 nm for rhodamine B.

### 2.6. Preparation of ESPNs

A mixture of 0%, 25%, 51%, and 75% WSPF (m/m, based on the total mass) was added to WF. The sample was then poured into a noodle machine and mixed thoroughly. Following this, 18% water was added and mixed for 2 min. The settings for the extrusion process, provided by Anhui Prestige Machinery and Equipment, were configured with the following temperatures: 150 °C for the initial zone, decreasing to 145 °C for the second, and further to 140 °C for the third zone. The feeding rate was maintained at 8 rpm, while the extrusion rate was set at 56 rpm. The extruder nozzle opening is round, with a thickness of 0.5 mm and a diameter of 15 cm. The product cutting specifications are as follows: a width of 1.5 cm and a length of 15 cm, and then portioned into segments weighing 52 g each. These segments underwent drying at a temperature of 40 °C for 2 h, followed by 20 h of acclimation to ambient temperature before being packaged and preserved. Visual documentation of the extruded sweet potato noodles (ESPNs) was conducted within a compact photography enclosure. A subset of the samples underwent pulverization, passing through a 120-mesh sieve, and were subjected to freeze-drying for 24 h in preparation for further analysis.

### 2.7. Determination of Cooking Characteristics of ESPNs

Adapting the approach outlined by Ji et al. [16], we analyzed to ascertain the culinary properties of the noodles. The ideal cooking duration was determined by immersing 52 g of noodles in a thermos flask containing 1000 mL of boiling water and monitoring the process with a chronometer. Beginning at the 180 s mark, noodles were extracted every 5 s to assess their texture; the rehydration time was noted when the noodles lacked a distinct firm (white) interior. For assessing water absorption and cooking loss, a 5 g portion of the instant noodle sample (initial mass m_1_) was immersed in 150 mL of boiling purified water until it reached the rehydration time. Post-draining for 5 min, the noodles’ mass (m_2_) was documented. The noodles’ water absorption percentage was computed employing Equation (2). The noodle broth was transferred to a beaker and reduced to 20 mL. The beaker’s initial mass (m_3_) was ascertained, and then it was dried in a 105 °C oven to a constant weight, recording the final mass (m_4_). The cooking loss percentage was derived using Equation (3). Each sample was subjected to triplicate trials.

(2) $$\mathrm{Water}\;\mathrm{absorption}/\%=\frac{m_{2}-m_{1}}{m_{1}}\times 100\%$$

(3) $$\mathrm{Cooking}\;\mathrm{Loss}\;\mathrm{Rate}\;(\%)=\frac{m_{4}-m_{3}}{m_{1}}\times 100\%$$

### 2.8. Determination of Expansion Capacity of ESPNs

The thickness of the extruded noodles was measured using an electronic digital caliper (Model DWKC-2012, Delixi Group Co., Ltd., Hangzhou, China). This measurement was then divided by the thickness of the die to calculate the noodles’ expansion ratio [17].

Flat noodles were selected, and their length was standardized to 5 cm. Subsequently, their length, width, and thickness after soaking for the rehydration period were measured and multiplied to obtain the volume of the noodles. Record the rehydrated volume as V_1_ and the initial volume as V_2_. The water absorption expansion ratio of the noodles was calculated using Equation (4).

(4) $$\mathrm{Water}\;\mathrm{absorption}\;\mathrm{expansion}\;\mathrm{ratio}=\frac{V_{1}-V_{2}}{V_{2}}$$

### 2.9. Texture Properties of Cooked ESPNs

Adhering to the protocol established by Yao et al. [18] for assessing the mechanical attributes of ESPNs with a texture analyzer (TMS-PRO, FTC, Sterling, VA, USA), the noodles were immersed until they reached their ideal rehydration point and subsequently cooled down to ambient temperature. For the texture profile analysis, the central portion of the noodle was chosen and tested using a cylindrical probe measuring 38 mm in diameter. The test parameters were configured as follows: the height of the probe from the surface of the bench was 10 mm; the deformation level was fixed at 75%; the probe’s speed of travel was set to 60 mm/min; the initial force applied was 0.1 N; and the pause between measurements was set at 2 s. Under these conditions, the hardness, elasticity, adhesion, resilience, and chewability of the crusts were evaluated.

### 2.10. Sensory Evaluation ESPNs

The evaluation criteria were referenced from Ji et al. [16] with modifications. A ten-member evaluation team was established to systematically assess the color, taste, smell, form, and texture of the ESPNs. The evaluators were professionally trained and ranged in age from 20 to 30 years, consisting of five men and five women. The scores obtained were summarized and averaged to determine the final score for each sample. For the evaluation, 50 g of the noodle products were taken. The products were first assessed for color, appearance, and form. They were then rehydrated, removed, and placed into clean containers for tasting and scoring. The entire scoring process was completed within 15 min. The sensory evaluation table is presented in Table 1.

### 2.11. Scanning Electron Microscope (SEM) of ESPNs

The microstructure of the ESPNs was examined using an SU8100 scanning electron microscope (Hitachi, Tokyo, Japan). It was placed under the scanning electron microscope and analyzed at a voltage of 10 kV, with magnifications of 100× and 5000× for scanning and imaging [9].

### 2.12. Thermal Properties of ESPNs

A Perkin-Elmer DSC 8000 differential scanning calorimeter from Waltham, MA, USA, calibrated using indium standards, was employed to analyze the thermal characteristics of lyophilized noodle specimens. The experimental protocol involved precisely weighing 2 mg of the powdered specimen into aluminum pans, followed by the addition of deionized water to establish a 2:1 water-sample ratio. Prior to measurement, the sealed samples underwent a 24-h equilibration at 4 °C. The thermal analysis was conducted against an empty reference pan, with temperature increasing from 25 to 150 °C at 5 °C/min [19].

### 2.13. X-Ray Diffraction (XRD) of ESPNs

The noodle’s crystallographic configuration was examined with a Bruker D8 Advance X-ray diffractometer, supplied by Bruker AXS GmbH, Karlsruhe, Germany. Following the procedures detailed by Li et al. [20], the analyses were conducted on samples that had been ground into powder form at room temperature, with a scanning velocity of 2 °/min across a 2θ diffraction range of 4° to 40°. The degree of relative crystallinity was then determined employing MDI Jade 6 software from Materials Data Inc., located in Livermore, CA, USA.

### 2.14. Fourier Transform Infrared Spectroscopy (FTIR) of ESPNs

The samples and potassium bromide powder were compressed into compact flakes approximately 0.5 mm thick, in a mass ratio of 1:50. The flake samples underwent spectral examination with a Fourier transform infrared (FTIR) spectrometer model Bruker VERTEX 70 from Thermo Fisher Scientific, Waltham, MA, USA, operating over a wavenumber range from 4000 to 400 cm^−1^ with a resolution setting of 4 cm^−1^. A total of 64 spectral acquisitions were performed, employing potassium bromide as the reference material, and the spectral background was subsequently removed, as reported by Liu et al. [21]. The spectral data underwent automatic baseline adjustment and Fourier deconvolution in the region of 1200–800 cm^−1^, facilitated by Omnic 8.2 software, also from Thermo Fisher Scientific, Waltham, MA, USA. Deconvolution settings included a half-width peak of 24.0 cm^−1^ and a resolution enhancement multiplier of 2.2. For the assessment of the short-range order within ESPNs, the infrared absorbance ratios between the wavenumbers 1043 and 1020 cm^−1^ (denoted as R_1043/1020_), and from 1020 to 993 cm^−1^ (denoted as R_1020/993_) were determined following the guidelines set forth by Yang et al. [22].

### 2.15. Statistical Analysis

Statistical processing of the data was carried out using SPSS software, version 26, from IBM, Armonk, NY, USA, and the outcomes are depicted as the average ± standard deviation. A unidirectional ANOVA was executed, supplemented by Duncan’s new multiple-range test, for evaluating variances between the sample groups at a significance threshold of *p* < 0.05. Visualizations of the data were crafted with OriginPro 2024b software by OriginLab, based in Northampton, MA, USA. Experiments were repeated three times for each measurement, except when indicated otherwise.

## 3. Results and Discussion

### 3.1. Starch Content of WSPF and WF

It was determined that WF contained 70.32% total starch, with linear amylose accounting for 25.34% of this total. Conversely, WSPF contained 69.7% total starch, with linear amylose comprising 20.57%. The free amylose content of WSPF was 24.17%.

### 3.2. The Mixing Behavioral Characteristics Analysis of Doughs

The Mixolab Mixing Tester was employed to assess the rheological characteristics of dough throughout the mixing process at a stable temperature and continuous heating and cooling. Several key indicators are analyzed concerning the past literature [23,24]. The entire testing process can be divided into five distinct stages: dough formation, protein weakening, starch pasting, amylase activity, and starch retrogradation. Among these stages, the dough behavior in the first two stages characterizes the powder properties. Table 2 presents the findings related to the properties of the powder as well as the thermo-mechanical characterization of the dough. Referencing Table 2, it is evident that the dough’s W_abs_ escalated with the incorporation of WSPF. This enhancement is likely due to the abundance of free starch, dietary fiber, and non-starch polysaccharides inherent in WSPF. The addition of WSPF resulted in a reduction of gluten protein concentration within the blended flour, promoting the swift establishment of gluten’s signature three-dimensional network [25]. As a result, there was a notable reduction in both the DDT and DST with the increased presence of WSPF.

**Figure 1:**
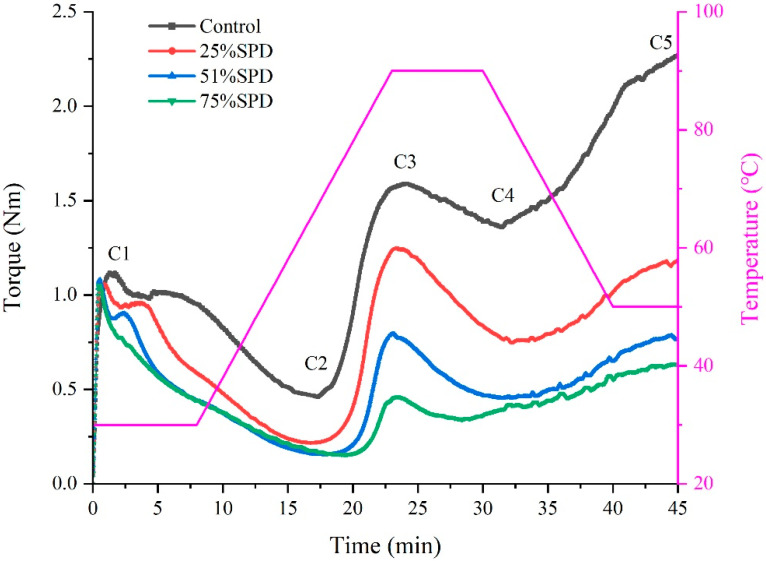
Mixolab profile of the SPDs.

Figure 1 displays the outcomes from the mixing analysis of SPD. Torque readings reflect the dough’s response to thermal and mechanical processes. C1–C2 indicate the extent to which mechanical and thermal forces weaken the protein network, while α represents the rate of weakening of the protein network. As the proportion of WSPF rose, the absolute *α* value progressively diminished, signifying WSPF’s role in slowing the degradation of the gluten matrix. The addition of WSPF to the dough led to a significant enhancement in dough weakening, as evidenced by a notable increase in C1–C2 values. This implies a reduced resistance to mechanical stress during mixing. The parameter C3 represents the pasting properties of starch within the dough, while *β* denotes the slope of the curve between C2 and C3, which indicates the rate of starch pasting. A decline was observed in both the C3 and *β* parameters for the reconstituted dough, likely due to the depletion of long-chain starch molecules in WSPF, consequently lowering the peak viscosity. The ratios C4/C3 and C5–C4 correspond to the cooking stability and retrogradation aging characteristics of starch pasting, respectively. In comparison to the control group, the WSPF led to a decline in the cooking stability of the dough (decrease in C4/C3 value); however, the 75%SPD was significantly greater than that of the initial two groups. The decrease in C5–C4 values indicates that WSPF reduces the retrogradation characteristics of s-pasted starch. This enhancement in stability may be linked to the modification of starch in the heat-treated WSPF, which increases viscosity and facilitates the gradual replacement of the gluten network structure by the starch gel structure, thereby improving cooking stability [9]. The parameter γ, representing the slope of the curve between C3 and C4, indicates the rate of amylase hydrolysis of starch. The decrease in the *γ*-value indicates that the addition of WSPF substantially reduces the rate of amylase hydrolysis in the reconstituted dough. In summary, the incorporation of WSPF at levels below 51% adversely affected the flour properties and thermo-mechanical characteristics of wheat dough. Specifically, dough stability decreased, and resistance to mechanical mixing was diminished. Conversely, when WSPF was added at a level of 75%, dough stability remained relatively high, indicating that 75% WSPF is more suitable for use in reconstituted doughs.

### 3.3. Rheological Properties Analysis of Doughs

**Figure 2:**
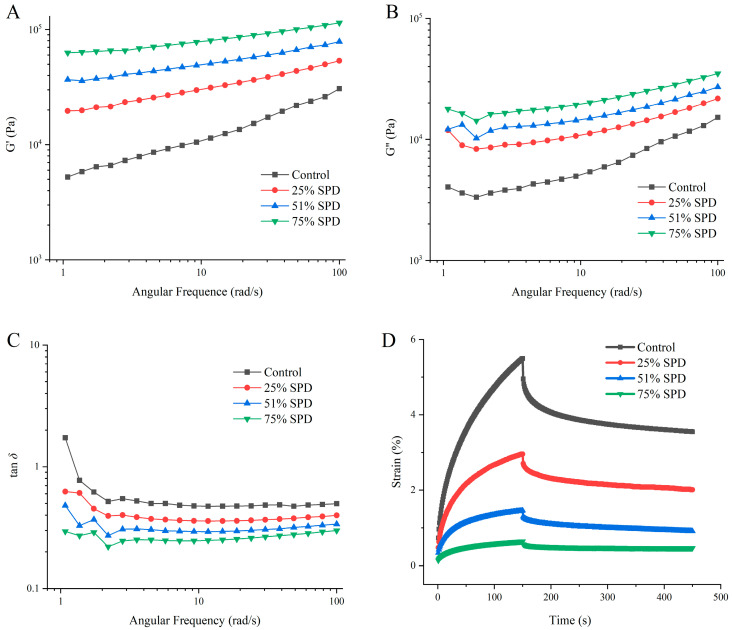
Rheological properties of SPDs (**A**–**D**) represent G′, G″, tan *δ*, and creep–response curves in that order.

Both storage modulus (G′) and loss modulus (G″) exhibited positive frequency dependence across the tested range (0.1–100 rad/s). The storage modulus G′ represents the elastic component of the dough’s viscoelastic behavior, corresponding to its capacity for energy storage during oscillatory deformation. In contrast, G″ characterizes the viscous response, reflecting the extent of energy dissipation throughout the deformation cycle. The rheogram of the dough is presented in Figure 2. The data illustrate that G′ consistently exceeds G″ throughout the frequency sweep (Figure 2A,B), suggesting that the dough composed of a mixture of WSPF and WF possesses solid-like elastic properties. The dough incorporating WSPF exhibited elevated values of both storage (G′) and loss (G″) moduli relative to the control sample, with an increasing tendency observed for both parameters. Such rheological enhancement suggests that thermally processed WSPF potentially acts as a hydrocolloid system, creating a protective matrix around dispersed particles, which consequently strengthens the dough’s structural integrity and elastic properties [14]. At a constant frequency, the addition of WSPF resulted in increased values for both the G′ and G″, with viscoelasticity also showing a tendency to rise. This rheological behavior could be explained by molecular interactions, specifically the formation of cross-linkages between WSPF starch molecules and WF protein components.

The phase angle tangent (tan *δ*), defined as the loss modulus to storage modulus ratio (G″/G′), serves as a key parameter characterizing the dough’s viscoelastic balance. The tan *δ* remained below unity for all specimens, reflecting the dominance of elastic over viscous behavior. In the low-frequency region (*ω* < 2.2 rad/s), a negative correlation was observed between tan *δ* values and angular frequency (Figure 2C). This trend aligns with previous findings on thermally modified barley flour dough systems [26]. The observed reduction in phase angle tangent suggests enhanced elastic characteristics of the dough matrix. However, at frequencies greater than 2.2 rad/s, the tan *δ* values tended to increase with increasing frequency, particularly for 25%SPD. This suggests that the dough exhibits a certain resistance to deformation at lower oscillation frequencies; however, as the frequency increases, the dough system begins to flow, leading to an increase in viscosity. The phase angle tangent exhibited an inverse relationship with WSPF concentration at equivalent frequencies. This rheological modification likely stems from molecular structural transformations, encompassing elevated free starch content, conformational reorganization of amylose and amylopectin chains, and intensified starch–protein molecular associations [27].

The curve of G′ versus frequency in Figure 2A follows the power law equation, and the fitting results are presented in Table 3. The coefficient K serves as an indicator of dough strength, where higher K values correspond to enhanced structural integrity [28]. The exponent z quantifies the frequency dependency of the storage modulus, providing insights into the network’s molecular architecture. A zero z-value indicates a covalently cross-linked stable network structure, whereas positive z-values suggest physically bonded networks with reduced stability [29]. From Table 3, it is evident that z values ranging from 0.196 to 0.398 suggest that all dough samples exhibited physically linked unstable network structures. The incorporation of increasing WSPF concentrations led to a progressive reduction in z values relative to the control, suggesting enhanced network stability in SPDs. The elevated K coefficients observed in SPDs indicate that thermally processed WSPF contributes to the reinforcement of WF’s structural integrity and dimensional stability.

Figure 2D illustrates the creep recovery curve of the dough, and the specific values obtained from the measurements are presented in Table 3. The deformation resistance of dough systems can be evaluated through maximum creep compliance (J_max_), where lower J_max_ values indicate enhanced structural rigidity [6]. Zero-shear viscosity (*η*_0_) quantifies flow resistance, with dough flowability indicating the presence of dynamic network structures rather than permanent cross-links [30]. SPDs exhibited reduced maximum creep strain (*ɣ*) compared to the control, consistent with their decreased J_max_ and elevated *η*_0_ values documented in Table 3. These rheological characteristics suggest that thermally processed WSPF enhances the structural resistance to both deformation and flow behavior. This reinforcement mechanism can be attributed to WSPF’s elevated content of liberated starch molecules and dietary fiber components, which facilitate increased water retention capacity and the development of supplementary hydrogen-bonding networks within the dough matrix [27]. The low J_max_ (0.98 × 10^−5^ Pa^−1^) and high *η*_0_ (165.65 × 10^3^ Pa·s) of the SFDs contribute to its superior capacity for deformation and flow resistance, consistent with the previously observed higher K values.

The recovery phase analysis reveals that the ratio J_e_/J_max_ (relative elastic component) correlates with structural recovery capability, while J_v_/J_max_ (relative viscous component) reflects gas retention properties [29]. Compared to the control, 25%SPD demonstrated enhanced elastic recovery characteristics, evidenced by elevated J_e_/J_max_ values, coupled with reduced J_v_/J_max_ ratios. These findings suggest that heat-treated WSPF can enhance the dough’s recovery ability and improve its elastic properties. However, the incorporation of 75% WSPF levels resulted in enhanced viscous components, as indicated by increased J_v_/J_max_ values. The findings demonstrate that WSPF supplementation effectively promotes dough network elasticity and structural stability.

### 3.4. CLSM Analysis of Doughs

**Figure 3:**
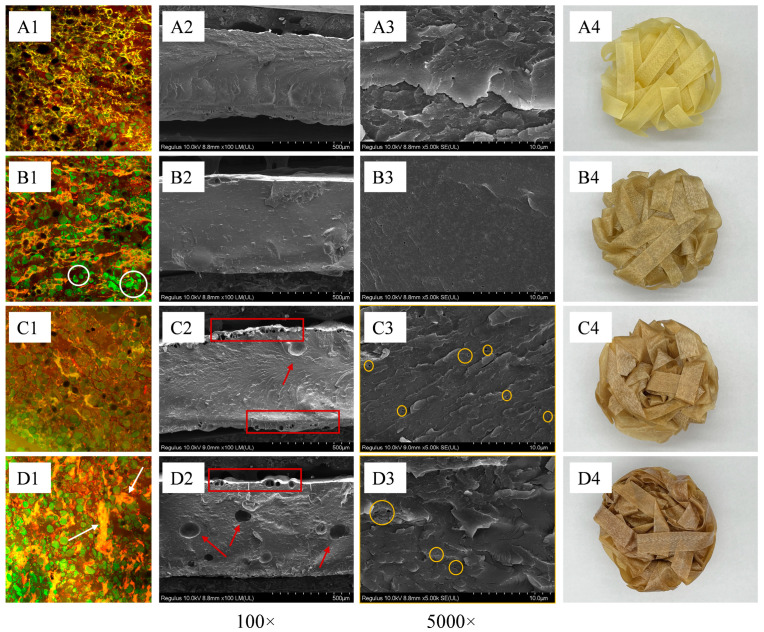
CLSM and SEM images of different dough and noodle samples. The (**A**–**D**) represents the control, 25%, 51%, and 75%, respectively; Subscripts 1–4 represent CLSM images of doughs, SEM 100× images of noodles, SEM 5000× images of noodles, and noodles images, respectively.

The primary structure that influences the quality of dough is the gluten network, which is interspersed with starch. The strength and toughness of this network directly affect the hardness and elasticity of noodles [31]. Fluorescein isothiocyanate and rhodamine B can bind to starch and protein, appearing green and red, respectively [15]. This allows for clear visualization of the cross-linking between gluten proteins and starch using laser confocal microscopy, thereby providing a more intuitive understanding of the changes in certain macroscopic properties of processed products made from WSPF. The microstructure of the dough is illustrated in Figure 3. In the control group, it can be observed that starch and protein are tightly bound to one another. However, with the addition of 25% WSPF, the starch granules become more pronounced and partially detach from the gluten network (as shown by the circles in the figure). The network structure of the 51%SPD becomes less distinct, while the starch gel structure becomes increasingly evident. An increase in the area of the red zone and the phenomenon of agglomeration were observed in 75%SPD (as indicated by the arrows in the figure) due to the inability of the proteins in WSPF to form a stable three-dimensional structure through disulfide bonds, as is the case with WF. Instead, these proteins were tightly bound to starch and other substances, preventing the gluten network from effectively enveloping the starch as the content of WSPF increases.

### 3.5. Cooking Characteristics of Noodles

**Figure 4:**
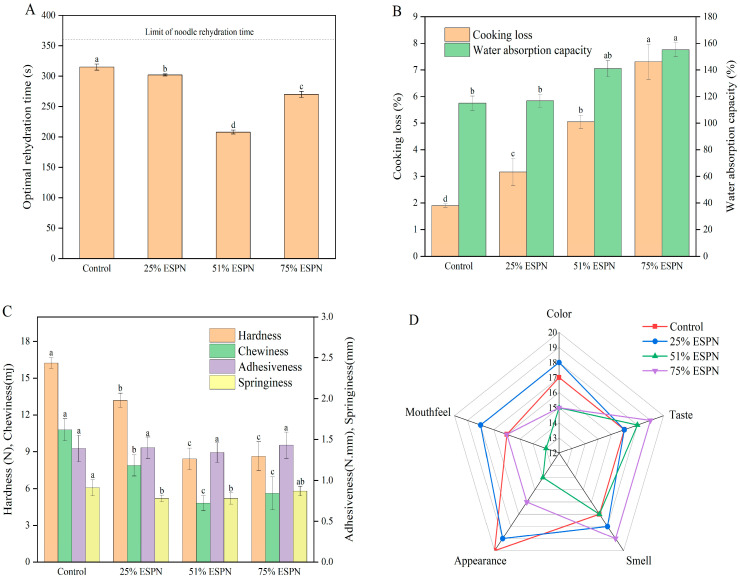
Optional rehydration time (**A**), cooking loss and water absorption (**B**), textural properties (**C**), and sensory scores (**D**) of different noodle samples. Different letters in the same index indicate significant differences (*p* < 0.05).

The rehydration time is a crucial indicator of the cooking quality of instant noodles. The cooking characteristics of the noodles are presented in Figure 4. Specifically, the rehydration times for Control, 25%ESPN, 51%ESPN, and 75%ESPN were 315 s, 300 s, 210 s, and 270 s, respectively (Figure 4A). The rehydration time of the instant noodles was influenced by the rate of complete gelatinization of starch following water absorption. On one hand, WSPF is gluten-free, and its incorporation into wheat dough disrupts the gluten network during the mixing process. When the proportion of WSPF is less than 51%, the starch that remains uncoated by protein becomes more pasty during extrusion, resulting in a finished product that is more prone to water absorption, thereby reducing the rehydration time [32]. On the other hand, the starch in cooked WSPF exhibits strong gelation properties, and the dietary fiber present in WSPF is abundant in hydrophilic groups, which absorb more water. Consequently, when the proportion of WSPF exceeds 51%, the starch gelation in the dough surpasses that of the gluten network, leading to an increased rehydration time for the noodles [9]. Notably, none of the rehydration times for sweet potato instant noodle crusts exceeded the 6-min rehydration time for non-fried instant noodles.

The water absorption of noodles increased from 115.02% to 155.24% as the proportion of WSPF was raised from 0% to 75% (Figure 4B). Concurrently, the cooking loss rate rose from 1.9% to 7.3%. This increase in water absorption can be attributed to the high dietary fiber content in WSPF. Additionally, the disruption of the gluten structure in the wheat dough led to the exposure of uncoated starch. When the dough was extruded and subsequently cooked, the starch rehydrated excessively, resulting in increased water absorption and swelling. This phenomenon also contributed to the elevated cooking loss rate of the noodles [33]. The cooking loss of 75%ESPNs did not exceed the maximum cooking loss of 12% observed in high-quality noodles, demonstrating that these noodles possess good cooking quality [34].

### 3.6. Expansion Capacity of ESPNs

The results of the expansion capacity of the noodles are presented in Table 4. The study indicated that the expansion ratio of the noodles initially decreased and then increased with the addition of WSPF. Notably, the water absorption expansion ratio of the noodles increased significantly (*p* < 0.05). The expansion capacity was consistent with the observations made using SEM, which revealed the presence of holes within the 75%ESPN, indicating some degree of swelling. Furthermore, the results of water absorption expansion ratio aligned with the findings on water absorption, which also increased with the addition of WSPF, resulting in a greater expansion volume of the noodles.

### 3.7. Texture Properties of Noodles

Holistic texture analysis is a testing method that simulates the chewing motion of the human body by extruding the sample twice with a probe. This process converts the abstract taste of the substance into concrete values, making the sensory indices more intuitive and tangible. High-quality instant noodles should exhibit a balance of moderate softness and firmness after rehydration, along with high elasticity, chewiness, and low viscosity [35]. The textural properties of ESPNs are presented in Figure 4C. With the increasing proportion of WSPF, the hardness, springiness, and chewiness of the ESPNs showed a significant decline, whereas adhesion levels remained relatively stable. The increased hardness observed in wheat noodles can be attributed to the robust structure and rigidity of the gluten network formed during cooking. Conversely, the addition of WSPF contributed more fiber and decreased the gluten content in the instant noodles, resulting in reduced density and lower measurements for various textural attributes. No significant differences were noted between the 75%ESPN and 51%ESPN samples, likely due to the improved starch gel formation induced by heat treatment, which corresponds with the results concerning cooking characteristics [36].

### 3.8. Sensory Analysis

The sensory evaluation assessed five aspects of ESPNs: color, taste, smell, mouthfeel, and appearance, with the results presented in Figure 4D. The total sensory scores for the four samples were 87, 90, 77, and 86, respectively. The radar chart indicates that 25%ESPN sensory acceptance was the highest, as all five evaluative indices received better scores. However, when the addition reached 51%, the color of the noodles darkened, and both the appearance and taste scores began to decline. Additionally, the noodles exhibited increased viscosity and a deteriorated chewing texture, resulting in lower acceptability for the 51%ESPN sample. Although the 75%ESPN sample was also darker, it possessed a rich sweet potato flavor, and its taste and appearance were superior to those of the 51%ESPN sample, leading to a higher total sensory score. This suggests that the addition of an appropriate amount of WSPF (either 25% or 75%) can enhance the quality of the noodles, imparting a sweet potato aroma, unique color, and satisfactory texture.

### 3.9. Microstructure of Different Noodles

From the SEM image at a magnification of 100× (Figure 3(A2,D2)), it was observed that the structure of the extruded instant noodles was compact. The high temperature and screw extrusion process rendered the starch pasty, resulting in the loss of the gluten network structure in the noodles. In comparison to the control group, holes were present at the edges of the ESPN cross-section as well as within the interior (as indicated by the arrows and circles in the figure). This phenomenon became more pronounced with an increased addition of WSPF. The increase in the number of holes contributed to a looser structure of the noodles upon water absorption, allowing the edge structure to easily disintegrate into the soup, thereby enhancing the mixing rate. This observation explains the finding that the cooking loss rate of the noodles was directly proportional to the amount of WSPF added. Observation of the 5000× image (Figure 3(A3,D3)) reveals that the 51%ESPN exhibits a higher number of holes in its microstructure, resulting in some disruption of structural continuity. In contrast, the 75%ESPNs display a denser and more continuous internal structure. When combined with the 200× image, it becomes evident that the 51%ESPN has more holes at the edges, which leads to a faster rate of starch water absorption and a shorter rehydration time for noodles. However, after rehydration, the edges of these noodles tend to detach, causing multiple noodles to adhere to one another, which negatively impacts sensory quality. Notably, the 75%ESPN has fewer holes at the edges compared to the 51%ESPN, and there is no adhesion observed after rehydration.

### 3.10. Thermal Properties of Noodles

The thermal–moisture conditions during extrusion induce molecular transformations in noodle components, characterized by protein cross-linking reactions and starch gelatinization phenomena. The thermal transition parameters, determined via DSC analysis and presented in Table 5, revealed significantly elevated onset (To), peak (Tp), conclusion (Tc), temperatures, and enthalpy changes (Δ*H*) in ESPNs relative to the control sample, with maximum increments observed in 51%ESPN. Δ*H* is correlated with the degree of structural ordering [37]. Mohamed and Rayas-Duarte [38] demonstrated that starch gelatinization behavior is substantially modulated by the presence of non-starch constituents within the dough matrix. Thermal processing during extrusion induced rapid moisture reduction in noodle matrices. Concurrently, the non-starch polysaccharides and dietary fiber components from WSPF interfered with intermolecular hydrogen bonding of starch, promoting enhanced starch–protein and starch–lipid complexation [39]. This molecular reorganization necessitates elevated thermal energy input for complete starch crystal transformation.

### 3.11. XRD Analysis of Noodles

**Figure 5:**
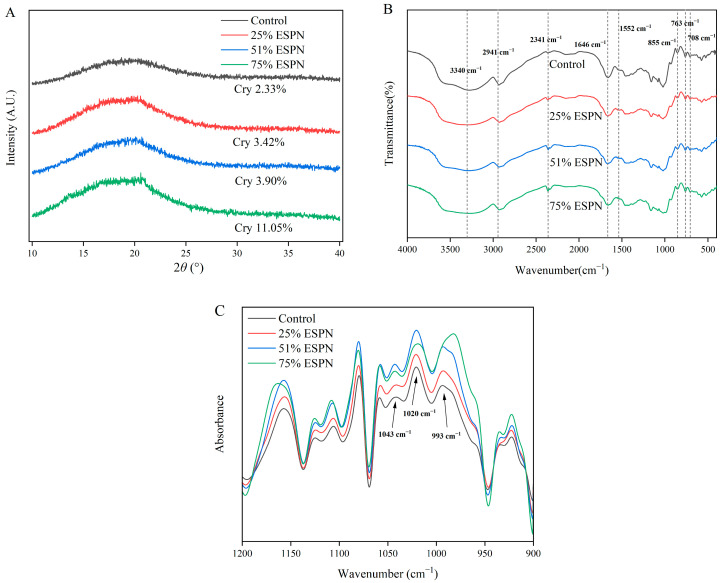
XRD patterns (**A**), FTIR spectra (**B**), and deconvolved IR spectra between 1200 and 900 cm^−1^ (**C**) of different noodle samples.

Figure 5A depicts the XRD analysis, which is employed to identify the crystalline nature and degree of crystallinity within the noodles. In the process of noodle extrusion, starch undergoes gelatinization due to exposure to high temperatures and pressures, causing a breakdown of the starch granules’ orderly structure and resulting in a less organized internal arrangement. The control sample exhibited a minimum crystallinity of 2.33%, whereas the crystallinity in the instant noodle samples enriched with WSPF showed a progressive rise, peaking at 11.05% in the 75%ESPN blend. This trend indicates that the incorporation of WSPF enhances the formation of an ordered crystalline structure in the extruded instant noodles. Cheetham et al. [40] reported that a higher content of straight-chain amylose correlates with lower starch crystallinity. The diffraction peaks of extruded WF noodles exhibited both low and wide dispersion peaks. The diffraction peaks at 2θ = 13° and 20° were progressively enhanced with increasing amounts of WSPF, indicating that these peaks are generally associated with straight-chain starch and lipids [5]. The elevated temperatures during the extrusion process disrupted the double-helix structure of branched-chain starch, allowing free lipids to form complexes with the leached straight-chain starch molecules [41].

### 3.12. Analysis of FTIR Experiment Results

Figure 5B shows that each sample displays distinctive absorption bands related to carbohydrates spanning from 4000 to 500 cm^−1^. The O-H vibration in starch generates a wide and strong infrared absorption band in the samples, extending between 3700 to 3000 cm^−1^, as cited in Li et al. [42]. Importantly, the O-H peak is consistently observed at approximately 3340 cm^−1^, suggesting that the energy associated with the O-H bond is relatively stable, in line with Yang et al. [43]. Moreover, the absorption bands at 2941 cm^−1^ and 2341 cm^−1^ are associated with the asymmetric stretching of -CH₂ and C-H bonds in linear starch molecules, respectively, as per Dar et al. [44]. The protein’s C=O bond stretching vibration is marked by the band at 1646 cm^−1^, aligning with the amide I band. This band also signifies the H-O-H bending mode of inbound water, indicating the involvement of both inter- and intramolecular hydrogen bonds. The band at 1552 cm^−1^ is linked to the N-H bending and C-N stretching in proteins, which is associated with the amide II band, as Zhang and Chen [9] have detailed. The distinctive peak wavenumbers for the pyranose ring’s backbone in the starch molecule are identified at 855 cm^−1^, 763 cm^−1^, and 708 cm^−1^, according to Dar et al. [44].

The detailed IR spectra for the noodle samples, depicted in Figure 5C, focus on the wavenumber range from 1200 to 900 cm^−1^. The spectral peaks in this region are characteristic of the unique ‘fingerprint’ zone for carbohydrates, which includes vibrations such as C-O, C-C, and C-O-H stretching, along with C-O-H bending vibrations [43]. Notably, the bands at 1043 cm^−1^ and 1020 cm^−1^ signify the structured and disordered starch molecular conformations, respectively. Moreover, the band at 993 cm^−1^ is associated with C-O-H bending, highlighting the relationship between water and starch molecules [45]. The ratios of 1040/1020 cm^−1^ and 1020/993 cm^−1^ serve as indicators of starch’s internal order and its interaction with water [46]. Table 5 shows that the R_1043/1020_ ratio for ESPNs increased markedly, while the R_1020/993_ ratio decreased significantly when compared to the control group. This suggests a more structured formation of starch at a local level in WSPF during the extrusion process, reducing its interaction with water [47]. Capron et al. [48] observed that the R_1020/993_ ratio is closely related to the crystallinity indicated by XRD data, with a higher R_1020/993_ ratio indicating reduced crystallinity, consistent with the XRD findings discussed in this study.

## 4. Conclusions

In this study, we investigated the effects of WSPF on the pasting characteristics, mixing properties, rheological behavior, and microstructure of the mixed dough, as well as the rehydration characteristics, textural quality, sensory analysis, and structural properties of the noodles. The following conclusions were drawn: regarding pasting characteristics, the addition of WSPF delayed dough aging and reduced the viscosity of the system. In terms of flour properties and thermo-mechanical characteristics, the incorporation of WSPF decreased the stability of the dough and weakened its resistance to mechanical mixing; however, the starch pasting cooking and retrogradation stability were relatively high with a 75% addition. The G′ and G″ of the dough increased with the addition of WSPF, indicating a more compact and elastic dough structure. As the substitution of WSPF increased, protein aggregation occurred in the dough, and holes appeared on the cut surface of the noodles, resulting in an increased cooking loss rate. Sensory evaluation revealed that the ESPNs exhibited a sweet potato aroma and flavor. XRD and FTIR analyses indicated that the relative crystallinity and short-range ordered structure of the ESPN improved, which aligned with the findings of high thermal stability observed in DSC. However, further comparisons between untreated and heat-fluidized WSPF as ingredients are still necessary. The 75%ESPNs demonstrated superior sensory scores and textural properties. The storage characteristics will be investigated further in future research.

## Figures and Tables

**Table 1:** Sensory evaluation criteria for ESPNs.

Evaluation Index	Scoring Criteria	Score (Points)
Color (20 points)	The bright yellow color is uniform throughout.	17–20
	The color is fairly bright but uneven.	12–16
	The dull yellow color exhibits poor saturation.	1–11
Appearance (20 points)	The surface structure is finely organized, smooth, and flat.	17–20
	The surface structure is more finely organized.	12–16
	Surface roughness, swelling, and severe deformation.	1–11
Smell (20 points)	The rich flavor of wheat and potatoes	17–20
	There is no noticeable taste or odor.	12–16
	Offensive odor.	1–11
Taste (20 points)	Sweet mouthfeel and aftertaste.	17–20
	There is no off-flavor or faintly sweet aroma.	12–16
	Sour, bitter, or other undesirable flavors.	1–11
Mouthfeel (20 points)	Moderate bite strength.	17–20
	Slightly soft or slightly hard.	12–16
	The balance between softness and hardness is crucial.	1–11

**Table 2:** Dough mixing behavior of different doughs using Mixolab.

Samples	DDT (min)	DST (min)	W_abs_ (%)	C1–C2 (Nm)	C3 (Nm)	C4/C3	C5–C4 (Nm)	*α* (Nm.min^−1^)	*β* (Nm.min^−1^)	*γ* (Nm.min^−1^)
Control	1.290 ± 0.020 ^a^	6.700 ± 0.020 ^a^	58.970 ± 0.060 ^d^	0.650 ± 0.020 ^c^	1.630 ± 0.040 ^a^	0.863 ± 0.012 ^a^	0.860 ± 0.050 ^a^	−0.073 ± 0.002 ^d^	0.332 ± 0.004 ^a^	−0.052 ± 0.002 ^b^
25%SPD	0.870 ± 0.010 ^b^	4.120 ± 0.030 ^b^	63.500 ± 0.100 ^c^	0.830 ± 0.020 ^b^	1.260 ± 0.030 ^b^	0.601 ± 0.042 ^c^	0.460 ± 0.030 ^b^	−0.042 ± 0.002 ^c^	0.215 ± 0.004 ^b^	−0.085 ± 0.001 ^c^
51%SPD	0.550 ± 0.020 ^c^	0.720 ± 0.020 ^c^	69.030 ± 0.060 ^b^	0.930 ± 0.020 ^a^	0.810 ± 0.020 ^c^	0.559 ± 0.021 ^c^	0.320 ± 0.010 ^c^	−0.034 ± 0.002 ^a^	0.120 ± 0.002 ^c^	−0.051 ± 0.001 ^b^
75%SPD	0.490 ± 0.010 ^d^	0.580 ± 0.010 ^d^	72.070 ± 0.120 ^a^	0.910 ± 0.010 ^a^	0.470 ± 0.020 ^d^	0.754 ± 0.042 ^b^	0.280 ± 0.020 ^c^	−0.038 ± 0.001 ^b^	0.078 ± 0.002 ^d^	−0.023 ± 0.003 ^a^

Note: Control: wheat flour dough; 25%SPD, 51%SPD, and 75%SPD: dough with 25%, 51%, and 75% substitution of whole sweet potato flour, respectively; DDT: dough development time; DST: dough stability time; W_abs_: water absorption; C1–C2: the degree of weakening of the protein; C3: starch pasting characteristics; C4/C3: starch pasting cooking stability: C5–C4: the retrogradation characteristics of s-pasted starch; *α*: rate of weakening of the protein network by heat; *β*: rate of starch pasting; *γ*: rate of starch hydrolysis by amylase. Different superscript letters in the same row are significantly different (*p* < 0.05).

**Table 3:** Fitting parameters for dynamic frequency–sweep curves and creep–recovery curves of different doughs.

Samples	z	K × 10^3^ (Pa·s^z^)	Creep		Recovery		
			J_max_ × 10^−2^ (Pa^−1^)	*η*_0_ × 10^3^ (Pa·s)	J_e_ × 10^−2^ (Pa^−1^)	J_e_/J_max_ (%)	J_v_/J_max_ (%)
Control	0.40 ± 0.02 ^a^	2.26 ± 0.17 ^d^	8.39 ± 0.11 ^a^	12.45 ± 0.07 ^d^	2.99 ± 0.02 ^a^	35.60 ± 0.00 ^b^	64.40 ± 0.00 ^a^
25%SPD	0.22 ± 0.02 ^b^	7.09 ± 0.47 ^c^	3.04 ± 0.03 ^b^	43.50 ± 0.14 ^c^	1.42 ± 0.07 ^b^	46.60 ± 0.01 ^a^	53.40 ± 0.01 ^b^
51%SPD	0.21 ± 0.01 ^c^	9.65 ± 0.43 ^b^	2.21 ± 0.05 ^c^	61.50 ± 0.14 ^b^	0.77 ± 0.11 ^c^	34.62 ± 0.01 ^b^	65.38 ± 0.03 ^a^
75%SPD	0.20 ± 0.01 ^d^	13.30 ± 0.53 ^a^	0.98 ± 0.02 ^d^	165.50 ± 0.21 ^a^	0.27 ± 0.01 ^d^	27.44 ± 0.00 ^b^	72.56 ± 0.00 ^a^

Note: Control: wheat flour dough; 25%SPD, 51%SPD, and 75%SPD: dough with 25%, 51%, and 75% substitution of whole sweet potato flour, respectively; z and K: the fitted power law parameters; J_max_: the maximum creep compliance; *η*_0_: zero-shear viscosity; J_e_: steady-state compliance; J_e_/J_max_: relative elastic component; J_v_/J_max_: relative viscous component. Different superscript letters in the same column are significantly different (*p* < 0.05).

**Table 4:** Expansion capacity results of different noodles.

Samples	ER	Length (mm)	Width (mm)	Thickness (mm)	WAER
Control	1.08 ± 0.02 ^b^	50.78 ± 0.57 ^c^	18.44 ± 0.67 ^b^	1.02 ± 0.02 ^c^	1.35 ± 0.01 ^d^
25%ESPN	1.04 ± 0.03 ^c^	50.98 ± 0.74 ^c^	18.86 ± 0.45 ^b^	1.04 ± 0.03 ^b^	1.56 ± 0.04 ^c^
51%ESPN	1.00 ± 0.00 ^d^	51.14 ± 0.46 ^b^	18.20 ± 0.54 ^b^	1.08 ± 0.07 ^b^	1.78 ± 0.02 ^b^
75%ESPN	1.20 ± 0.02 ^a^	52.78 ± 0.12 ^a^	19.84 ± 0.76 ^a^	1.22 ± 0.020 ^a^	1.83 ± 0.04 ^a^

Note: Control: extruded wheat flour noodles; 25%ESPN, 51%ESPN, and 75%ESPN: extruded noodles with 25%, 51%, and 75% substitution of whole sweet potato flour, respectively; ER: expansion ratio; length, width, and thickness: noodle size after rehydration; WAER: water absorption expansion ratio. Different superscript letters in the same column are significantly different (*p* < 0.05).

**Table 5:** Thermal properties and FTIR deconvolution results of different noodles.

Samples	To (°C)	Tp (°C)	Tc (°C)	Δ*H* (J/g)	R_1043/1020_	R_1020/993_
Control	117.32 ± 0.20 ^c^	117.49 ± 0.25 ^c^	122.9 ± 0.27 ^c^	0.57 ± 0.01 ^c^	0.70 ± 0.03 ^c^	1.22 ± 0.05 ^a^
25%ESPN	123.38 ± 0.64 ^b^	123.66 ± 0.35 ^b^	124.59 ± 0.52 ^b^	0.77 ± 0.02 ^c^	0.73 ± 0.02 ^b^	1.16 ± 0.03 ^b^
51%ESPN	132.13 ± 0.32 ^a^	141.42 ± 0.16 ^a^	148.22 ± 0.29 ^a^	19.04 ± 0.5 ^b^	0.75 ± 0.02 ^b^	1.13 ± 0.02 ^c^
75%ESPN	131.12 ± 0.79 ^a^	141.2 ± 0.28 ^a^	148.01 ± 0.58 ^a^	22.6 ± 0.12 ^a^	0.78 ± 0.01 ^a^	1.01 ± 0.05 ^d^

Note: Control: extruded wheat flour noodles; 25%ESPN, 51%ESPN, and 75%ESPN: extruded noodles with 25%, 51%, and 75% substitution of whole sweet potato flour, respectively; To: onset temperature; Tp: peak temperature; Tc: conclusion temperature; Δ*H*: enthalpy; R_1043/1020_: the ratios of IR absorbance at 1043 to 1020 cm^−1^; R_1020/993_: the ratios of IR absorbance at 1020 to 993 cm^−1^. Different superscript letters in the same column are significantly different (*p* < 0.05).

## Data Availability

The original contributions presented in the study are included in the article, further inquiries can be directed to the corresponding author.
